# Supplementary figures and images for: Contribution of trans regulatory eQTL to cryptic genetic variation in C. elegans
Source: BMC Genomics. 2017 Jun 29;18:500. doi: 10.1186/s12864-017-3899-8 (PMC5492678; doi:10.1186/s12864-017-3899-8)

I

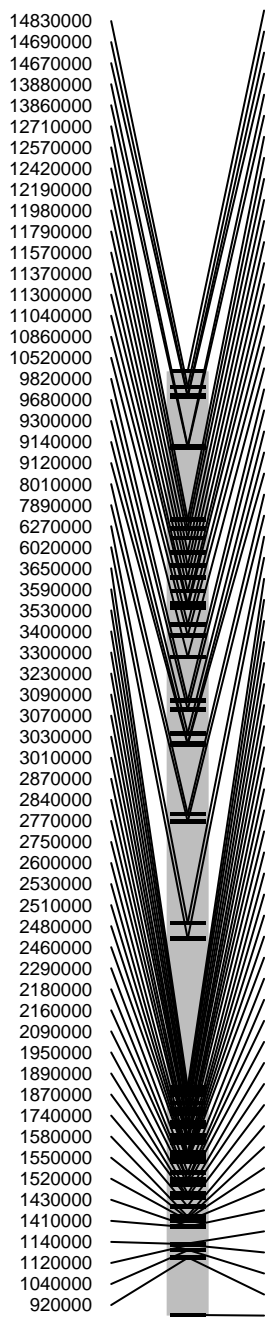

II

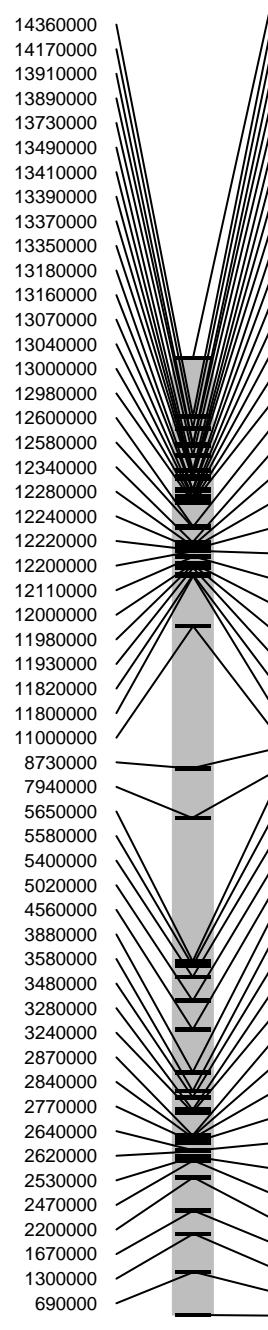

III

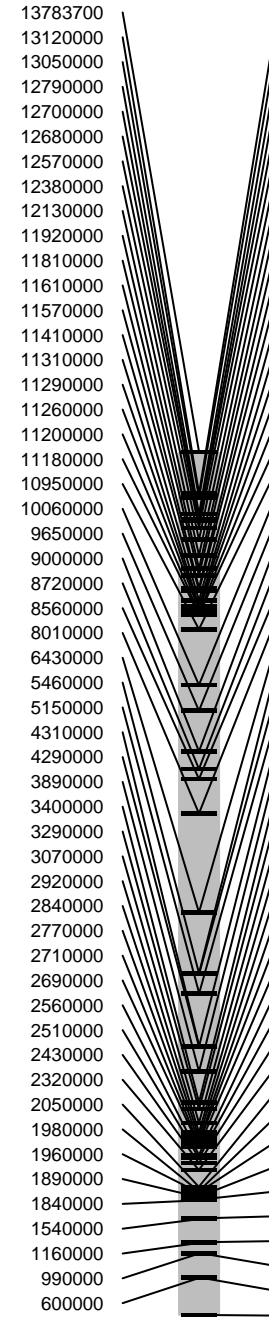

IV

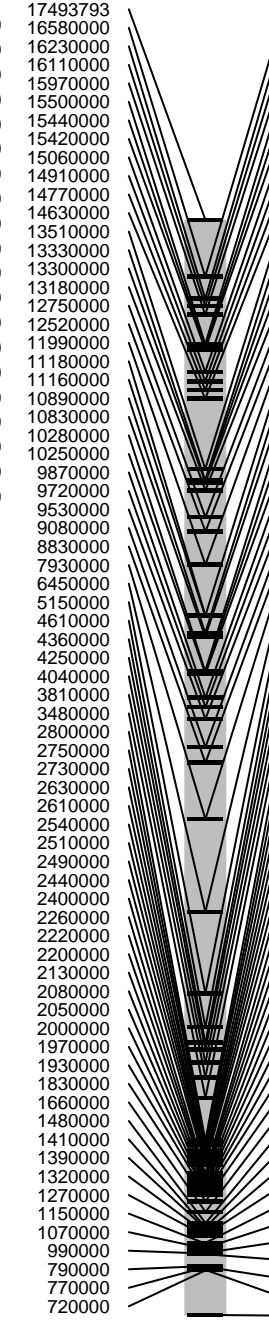

V

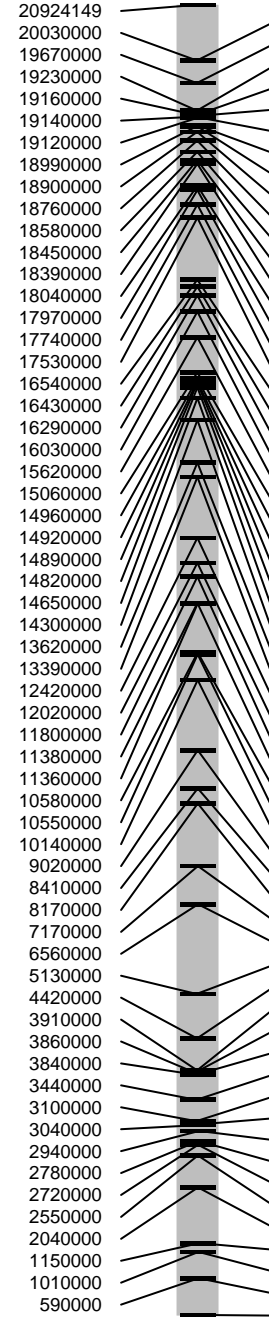

X

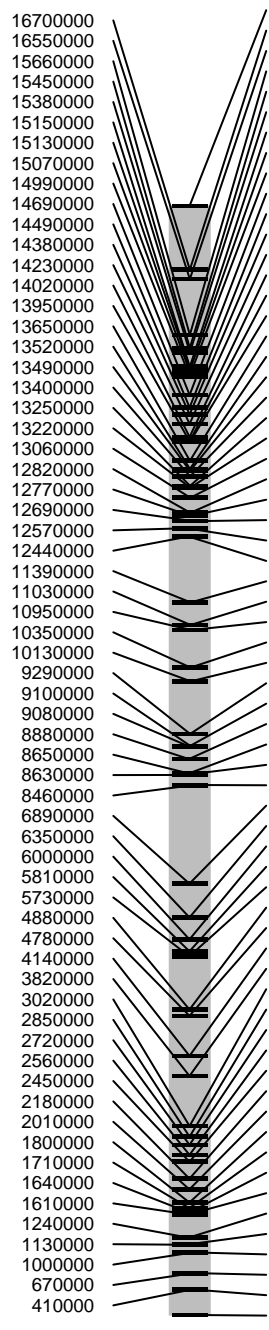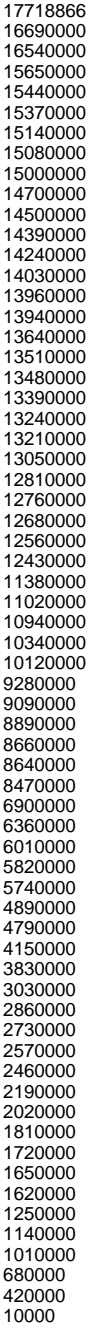

Supplement: Supplementary file 2 — A figure of the location of the 729 markers. The marker locations are plotted across the genome. Locations are based on WS256. (PDF 20 kb) [file 12864_2017_3899_MOESM2_ESM.pdf]

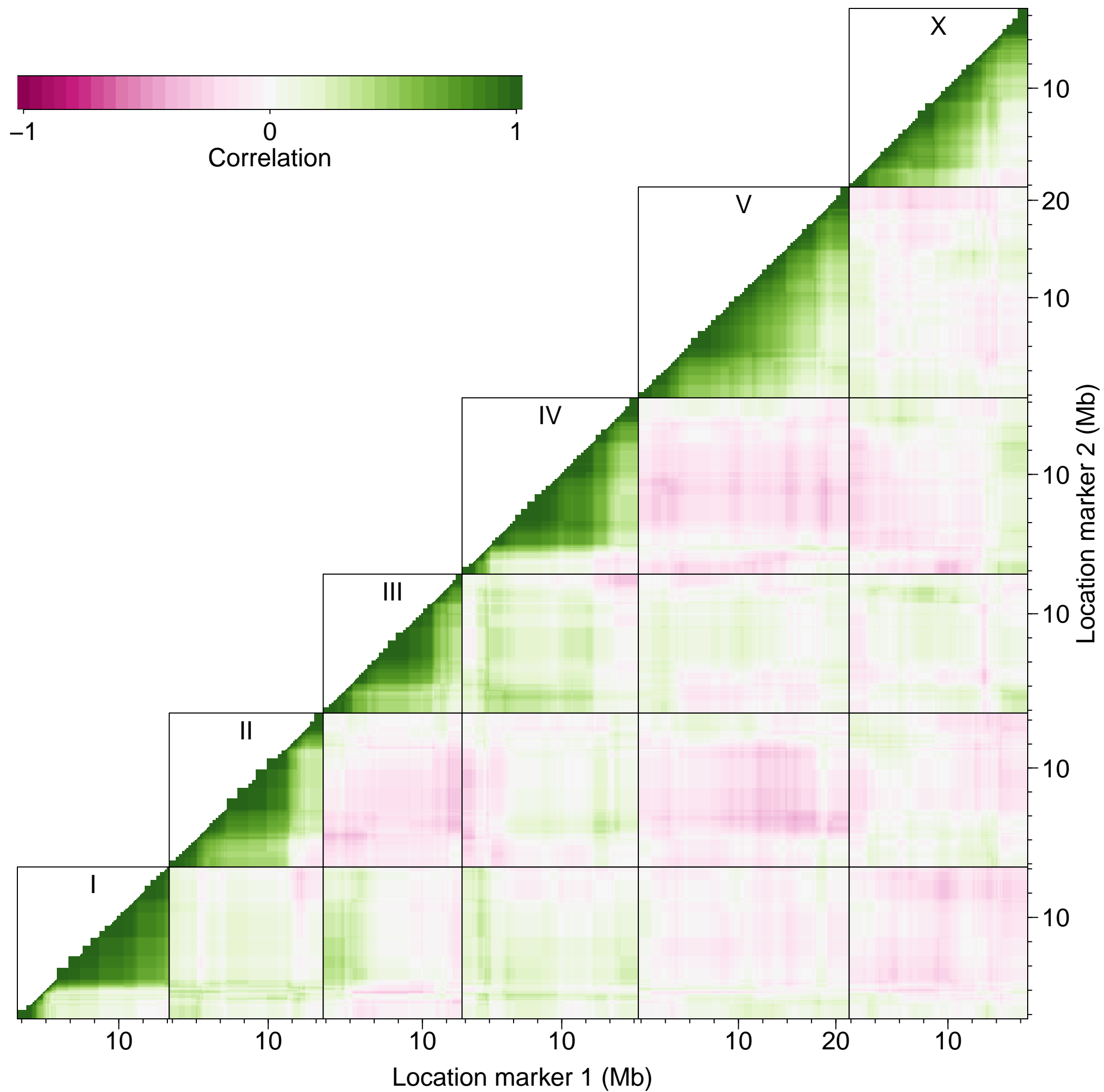

Supplement: Supplementary file 3 — A figure of the marker correlation analysis. Correlations between the 729 markers in the sequenced RIL population. The markers are plotted at their physical locations across the chromosomes. (PDF 1279 kb) [file 12864_2017_3899_MOESM3_ESM.pdf]

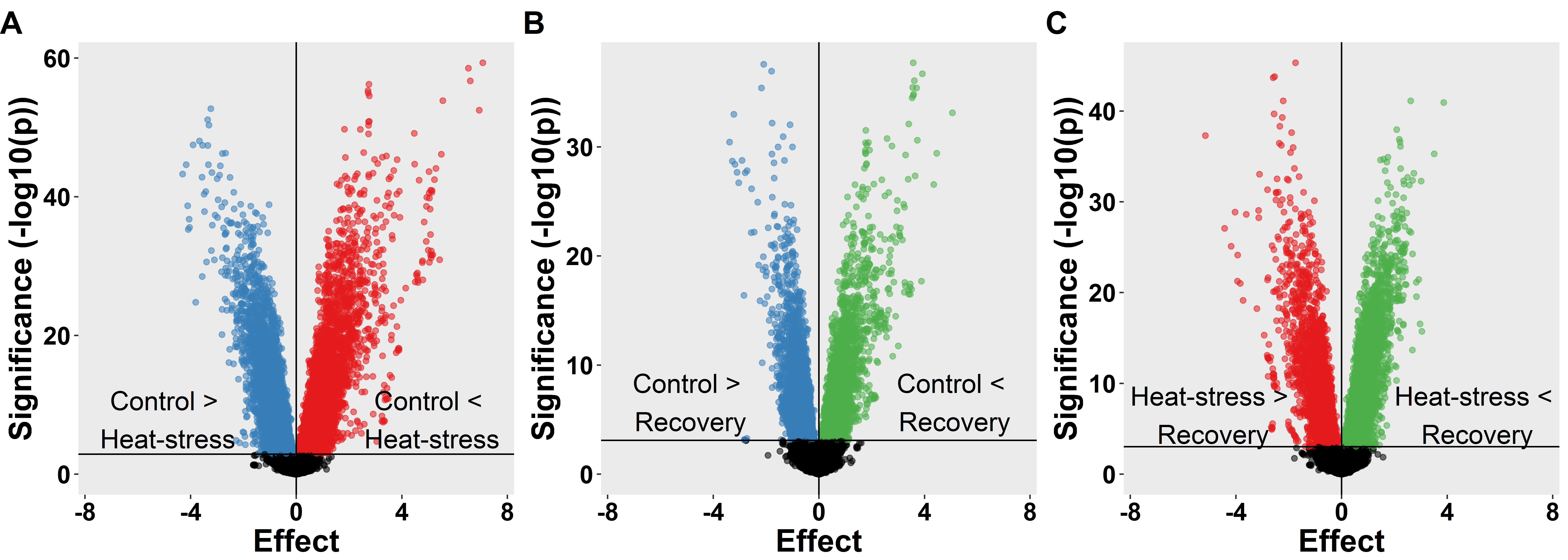

Supplement: Supplementary file 4 — Figure of the treatment comparisons. Volcano plots of the expression comparisons per treatment (n = 48 RILs per treatment). The horizontal line in the plots indicates the FDR = 0.05 threshold. The colored dots indicate spots that are significantly different between treatments. Blue indicates spots more highly expressed in the control treatment, red indicates spots more highly expressed in the heat-stress treatment, and green indicates spots more highly expressed in the recovery treatment. (A). The comparison between control and heat stress, threshold: -log10(p) ≥ 2.87. (B). The comparison between control and recovery, threshold: -log10(p) ≥ 3.09. (C). The comparison between heat stress and recovery, threshold: -log10(p) ≥ 3.02. (TIFF 1329 kb) [file 12864_2017_3899_MOESM4_ESM.tiff]

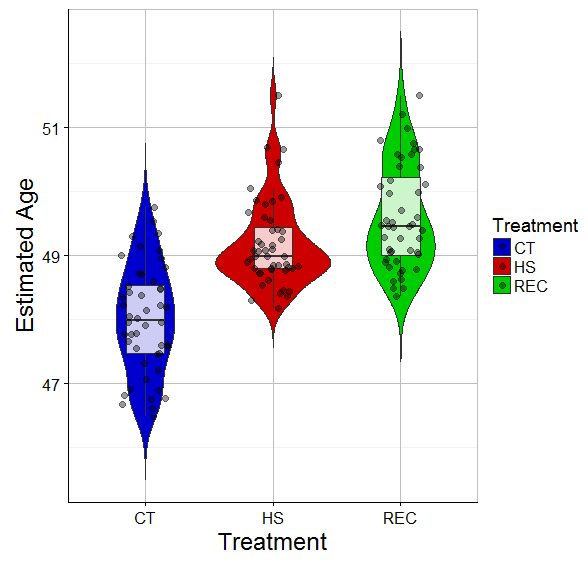

Supplement: Supplementary file 8 — Developmental ruler applied to the RIL populations. The age of the samples was estimated using the transcriptional ruler from [9], the reported ages are relative, where the average age of the control samples were set to 48 h. Each point represents one sample, whereof the relative age was determined by assessing the expression of about 100 genes. (TIFF 969 kb) [file 12864_2017_3899_MOESM8_ESM.tiff]

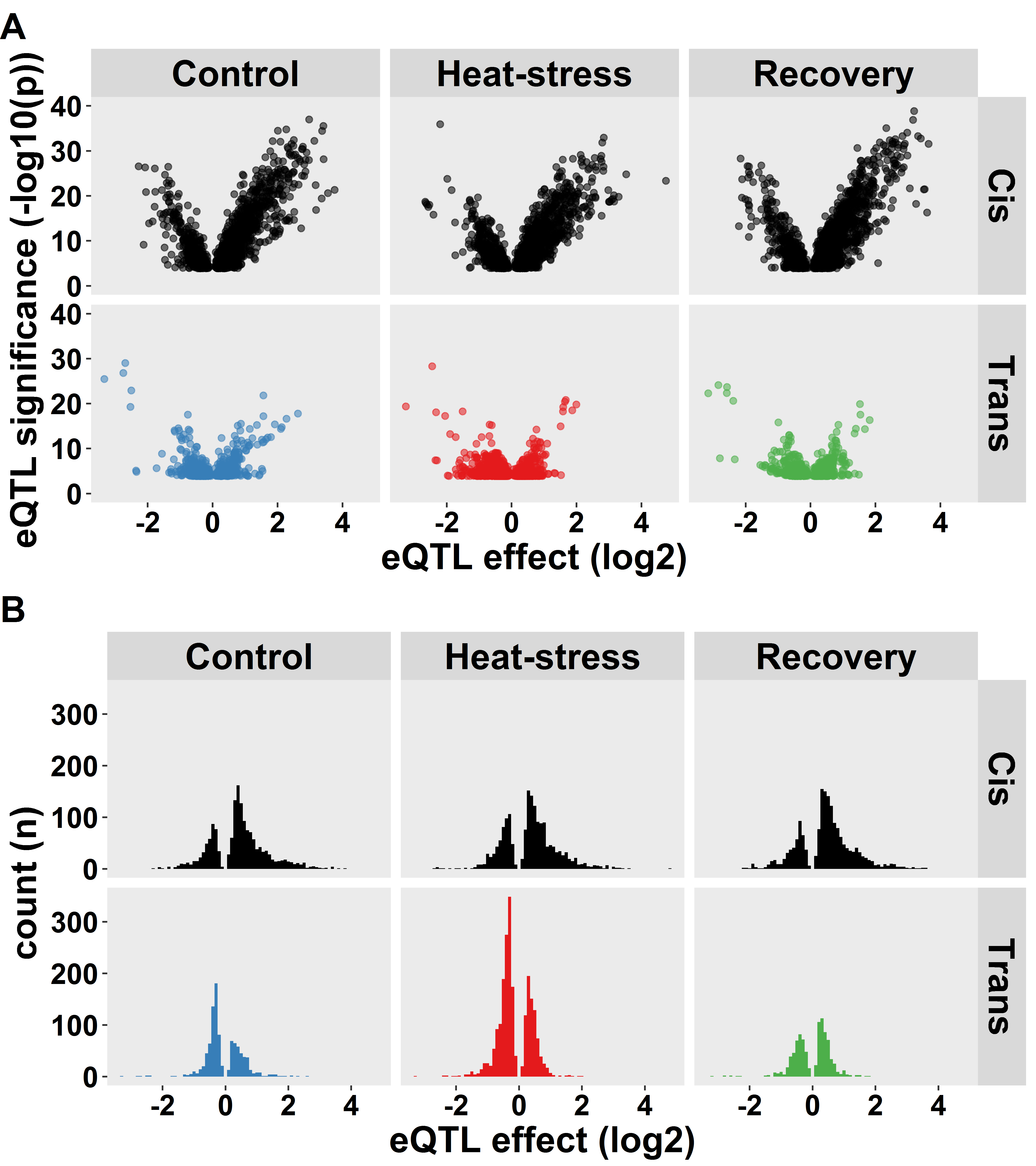

Supplement: Supplementary file 11 — Figure of eQTL effect distribution. (A) Volcano plots of the eQTL mapped per type (cis or trans) per treatment. On the x-axis the effect is plotted and on the y-axis the significance of the association is plotted (−log10(p)). Each dot represents a microarray spot and only the significant associations are shown (FDR ≤ 0.05, −log10(p) > 3.9 in all three treatments). (B) A histogram of the eQTL effect sizes, per type (cis or trans) per treatment. Again, the number of significantly associated spots are counted. (TIFF 738 kb) [file 12864_2017_3899_MOESM11_ESM.tiff]

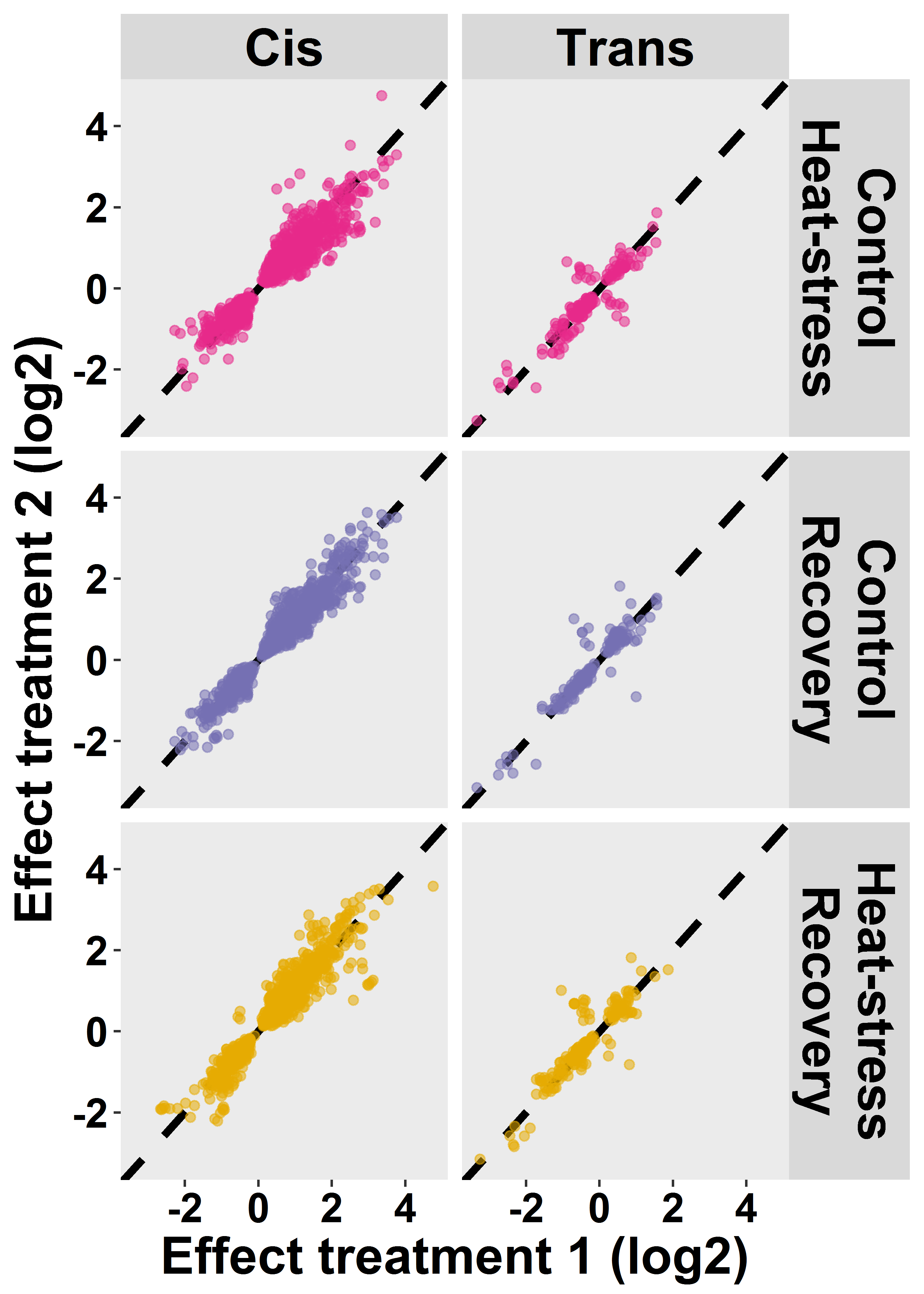

Supplement: Supplementary file 14 — Figure comparing allelic effects of genes with an eQTL between treatments. A scatter plot of the effects of the eQTL of genes with an eQTL found in multiple treatments. Each dot represents a spot. The Pearson correlation values between the different comparisons are: R = 0.94 and 0.91 for cis- and trans-eQTL in control versus heat stress, R = 0.96 and 0.93 for cis- and trans-eQTL in control versus recovery, and R = 0.94 and 0.89 for cis- and trans-eQTL in heat stress versus recovery. The striped diagonal lines are shown as an optical reference. (TIFF 454 kb) [file 12864_2017_3899_MOESM14_ESM.tiff]
