# Supplementary material for: Contribution of trans regulatory eQTL to cryptic genetic variation in C. elegans
Source: BMC Genomics. 2017 Jun 29;18:500. doi: 10.1186/s12864-017-3899-8 (PMC5492678; doi:10.1186/s12864-017-3899-8)
Supplement: Supplementary file 15 — Text detailing the calculations on overlap in cis- and trans-eQTL. (DOCX 23 kb) [file 12864_2017_3899_MOESM15_ESM.docx]

**Additional file 14: Supplementary text**

*Overlap in* cis-*eQTL over three treatments*

It was found that ~61% of the *cis*-eQTL occurred in more than one treatment. Based on permutated data (n = 10 permutations per treatment), the chance of overlap at the used thresholds was almost non-existent (0.2%). This was to be expected, as *cis*-eQTL were required to be found near the affected gene, which reduced the chance of a random association to result in a significant *cis*-eQTL.

On average, the *cis*-eQTL explained 52.1% of the variation, where *cis*-eQTL occurring in more than one treatment explained 57.7% of variation and *cis*-eQTL not occurring in multiple treatments explained 35.7% of variation (**Table S1**). By comparing the distribution of variation explained with the power analysis (**Table S1**), it was expected that 89.0% of *cis*-eQTL would be detected over multiple treatments. However, this would be an upper boundary, as we were likely to over-estimate the small effect sizes and the true eQTL distribution remains unknown.

| **Table S1: Variation explained by eQTL** | | | | |
| --- | --- | --- | --- | --- |
| eQTL type | Occurrence | Number (percentage)^1^ | Expected percentage^2^ | Average variation explained (R^2^) |
| *cis* | 1 treatment | 1280 (25.2%) | 11.0% | 0.357 |
|  | >1 treatment | 3799 (74.8%) | 89.0% | 0.577 |
|  | **total** | **5079** |  | **0.521** |
|  |  |  |  |  |
| *trans* | 1 treatment | 3472 (84.8%) | 26.4% | 0.320 |
|  | >1 treatment | 624 (15.2%) | 73.6% | 0.466 |
|  | **total** | **4096** |  | **0.342** |
| ^1^: number of spots with an eQTL  ^2^: based on comparison between the distribution of variation explained by the QTL with the power analysis | | | | |

It was likely that the eQTL detected in singular treatments consisted of three types: (i) false positives, (ii) small effect eQTL, and (iii) true treatment specific eQTL. Since *cis*-eQTL consistently showed the same effect (**Additional file 10**), it was to be expected that false positive eQTL were likely to show different effect directions across conditions. On the other hand, small effect eQTL were expected to show the same effect direction, but were not detected due to lack of statistical power. Based on these assumptions, we estimated that ~29% (186*2/1280) of the non-detected spots with an eQTL were false negatives and ~71% of these eQTL were undetected small effect *cis*-eQTL (**Table S2**). Thus, only a small number of *cis*-eQTL would be treatment specific.

| **Table S2: Effect directions of eQTL across treatments** | | | |
| --- | --- | --- | --- |
| eQTL type | Different effect directions^1^ | Number (percentage)^2^ | Average variation explained by QTL (R^2^) |
| *cis* | No | 4893 (96.3%) | 0.523 |
|  | Yes | 186 (3.7%) | 0.356 |
|  | **total** | **5079** | **0.521** |
| *trans* | No | 2717 (66.3%) | 0.357 |
|  | Yes | 1379 (33.7%) | 0.313 |
|  | **total** | **4096** | **0.342** |
| ^1^: at the same locus over all treatments  ^2^: number of spots with an eQTL | | | |

*Overlap in* trans*-eQTL over three treatments*

It was found that only ~14% of the genes with a *trans*-eQTL were detected in more than one treatment (15.2% of the spots). When comparing the mapped *trans*-eQTL with permutated data (n = 10 permutations per treatment), the chance to find the same gene with a *trans*-eQTL in a permutated treatment dataset was 0.2%. This led to an estimation that 3.3% of the 360 genes with a *trans*-eQTL detected in multiple treatments are false-positives. These false *trans*-eQTL occurring in more than one treatment were most likely to originate from a different chromosome, for which 9.6% *trans*-eQTL were expected to occur as false positives.

On average, the *trans*-eQTL explained 34.2% of the variation, which is far less than what we observed for the *cis*-eQTL. The *trans*-eQTL detected in more than one treatment explained 46.6% of the variation, whereas *trans*-eQTL detected in only one treatment explained 32.0% of the variation (**Table S1**). From this, we concluded that *trans*-eQTL with a large effect size were more likely to occur in multiple treatments. Yet, based on the distribution of the variation explained compared to the power analysis (**Table S1**), we expected to detect 73.6% of all the *trans*-eQTL in multiple treatments. This was under the assumption that the true QTL effect-size distribution was measured; the effect size was not over-estimated for the *trans*-eQTL. Because it was likely that we over-estimated the effect size, since the variation explained per *trans*-eQTL was relatively low for our statistical power (**Additional file 8**), this estimation corresponded to an upper limit and we were likely to detect less *trans*-eQTL in multiple treatments. However, as only 15.2% of the spots with a *trans*-eQTL were indeed found in multiple treatments, it seems unlikely that lack of statistical power is the cause for the majority of *trans*-eQTL to be environment specific.

In order to further substantiate this observation we used the observation that *trans*-eQTL display the same effect direction and size when present in multiple treatments (**Additional file 13B**). From this observation we predicted that if *trans*-eQTL are truly absent in one treatment, the effect directions for these *trans*-eQTL should be random. Whereas if a *trans*-eQTL is not detected due to lack of statistical power, the effect direction should be similar. We compared the variation explained and the effect direction at the locus where a *trans*-eQTL was mapped in one treatment (*e.g.* control), with the variation explained and effect direction at the same locus in the other treatments (heat-stress and recovery in this example). From this comparison we found that out of 4096 microarray spots with a *trans*-eQTL in one of the treatments. When we assume that *trans*-eQTL have the same effect direction across treatments, we find that at the same locus 1379 (33.7%) showed an effect opposite to the expected effect and 2717 (66.3%) showed a similar effect. Therefore, we estimated that 79.4% (1379*2/3472) of the spots with a *trans*-eQTL occurring in only one treatment represent cryptic genetic regulation.

*Overlap in* trans-*bands across treatments*

As a large proportion of the *trans*-eQTL occurred in *trans*-bands, and 13/19 identified *trans*-bands co-localize across treatments, we determined the overlap in affected eQTL. If *trans*-eQTL were indeed treatment specific, it was to be expected that also the *trans*-bands were (mostly) treatment specific. Therefore, the overlap in genes with an eQTL between *trans*-bands was calculated (**Table S3**).

| **Table S3: overlap in eQTL belonging to a *trans*-band.** | | | | | |
| --- | --- | --- | --- | --- | --- |
| Treatment 1  *trans*-band | Treatment 2  *trans*-band | eQTL 1 (n) | eQTL 2 (n) | Overlap^1^ | Significance^2^ |
| Control, I:3.5-4 | Heat-stress, I:2-3.5 | 18 | 480 | 1 (0.2%) | p = 0.871 |
| Control, I:3.5-4 | Recovery, I:1.5-3 | 18 | 197 | 1 (0.5%) | p = 0.399 |
| Heat-stress, I:2-3.5 | Recovery, I:1.5-3 | 480 | 197 | 4 (0.6%) | p = 1 |
| Control, V:11-11.5 | Recovery, V:11.5-12 | 23 | 17 | 4 (11.1%) | p = 1.93*10^-7^ |
| Heat-stress, III:0.5-2 | Recovery, III:2-2.5 | 54 | 41 | 2 (2.2%) | p = 0.051 |
| Heat-stress, IV:1-2.5 | Recovery, IV:1-2 | 244 | 31 | 22 (8.7%) | p = 3.20*10^-18^ |
| Heat-stress, IV:4-4.5 | Recovery, IV:4-4.5 | 30 | 19 | 3 (6.5%) | p = 4.88*10^-5^ |
| Heat-stress, V:1-3 | Recovery, V:2.5-3 | 125 | 13 | 1 (0.7%) | p = 0.126 |
| ^1^: overlap as a percentage of the number of unique genes covered by both *trans*-bands.  ^2^: of overrepresentation, as calculated by a hypergeometric test. | | | | | |

It was found that most *trans*-bands indeed were treatment specific. Most surprisingly, even the *trans*-band on chromosome I, which affected many genes in both heat-stress and recovery, was not the same *trans*-band in both treatments. Although 3 pairs of *trans*-bands were significantly overlapping, the actual number of overlapping genes was quite low.
